# Comparison of three common tonsil surgery techniques: cold steel with hot hemostasis, monopolar and bipolar diathermy

**DOI:** 10.1007/s00405-023-07892-3

**Published:** 2023-02-23

**Authors:** Jenny Christina Knubb, Jasmin Maria Kaislavuo, Henri Sebastian Jegoroff, Jaakko Matias Piitulainen, Johannes Routila

**Affiliations:** 1 Otorhinolaryngology, Faculty of Medicine, University of Turku, Kiinamyllynkatu 4-8, 20521 Turku, Finland; 2 Department of Otorhinolaryngology, Satakunta Hospital District, Satasairaala Central Hospital, Pori, Finland; 3 Division of Surgery and Cancer Diseases, Department of Otorhinolaryngology, Head and Neck Surgery, Turku University Hospital, Turku, Finland

**Keywords:** Tonsillectomy, Tonsillotomy, Postoperative hemorrhage, Postoperative bleeding, Risk factors

## Abstract

**Purpose:**

To analyze the risk of postoperative hemorrhage in tonsil surgery with different surgical methods, instruments, indications, and age groups. Monopolar diathermy compared to bipolar diathermy was of particular interest.

**Methods:**

The data from tonsil surgery patients were retrospectively collected between 2012 and 2018 in the Hospital District of Southwest Finland. The surgical method, instruments, indication, sex and age and their association with a postoperative hemorrhage were analyzed.

**Results:**

A total of 4434 patients were included. The postoperative hemorrhage rate for tonsillectomy was 6.3% and for tonsillotomy 2.2%. The most frequently used surgical instruments were monopolar diathermy (58.4%) cold steel with hot hemostasis (25.1%) and bipolar diathermy (6.4%) with the overall postoperative hemorrhage rates 6.1%, 5.9% and 8.1%, respectively. In tonsillectomy patients, the risk for a secondary hemorrhage was higher with bipolar diathermy compared to both monopolar diathermy (p = 0.039) and the cold steel with hot hemostasis technique (p = 0.029). However, between the monopolar and the cold steel with hot hemostasis groups, the difference was statistically non-significant (p = 0.646). Patients aged > 15 years had 2.6 times higher risk for postoperative hemorrhage. The risk of a secondary hemorrhage increased with tonsillitis as the indication, primary hemorrhage, tonsillectomy or tonsillotomy without adenoidectomy, and male sex in patients aged ≤ 15 years.

**Conclusion:**

Bipolar diathermy increased the risk for secondary bleedings compared to both monopolar diathermy and the cold steel with hot hemostasis technique in tonsillectomy patients. Monopolar diathermy did not significantly differ from the cold steel with hot hemostasis group regarding the bleeding rates.

## Introduction

Tonsil surgery is one of the most common ambulatory surgeries [1]. It is performed using different surgical methods, including extracapsular tonsillectomy (TE) and tonsillotomy (TT) with or without adenoidectomy [2]. Tonsil surgery is performed for several indications, the most frequent of which are recurrent and chronic tonsillitis and upper airway obstruction related to tonsil hypertrophy.

Postoperative hemorrhage is one of the most common complications in tonsil surgery often requiring medical intervention and can even be life-threatening [3]. Bleeding rates vary greatly among different studies from 0.3 to 15% due to different populations, study design and the definition of postoperative bleeding [4, 5].

In tonsil surgery, several different instruments are used both for dissection and for hemostasis. Dissection instruments can be roughly divided into cold and hot techniques according to the thermal effect. Knife, scissors, tonsil elevator and microdebrider belong to the cold reduction techniques and monopolar needle, bipolar scissors and forceps, coblation, radiofrequency, laser, argon plasma coagulation, thermal welding and ultrasound belong to the hot techniques [6].

Multiple studies recommend using cold techniques instead of hot instruments due to the increased risk of postoperative hemorrhage related to hot techniques [7–12]. Several of these studies have compared the cold technique to bipolar diathermy and coblation. However, clinical practice has evolved towards the use of hot techniques [7, 13]. Many surgeons have adapted hot instruments because they allow better control of bleeding during surgery and thus reduce intraoperative bleeding and shortening the operation time [14]. There is still a scarcity of knowledge on the risk of postoperative bleeding when using monopolar diathermy, even if monopolar diathermy is readily available in most operation rooms.

The aim of this retrospective study was to analyze the frequency of postoperative hemorrhage with different surgical methods and surgical instruments. Monopolar diathermy compared to bipolar diathermy was of particular interest. In addition, the association of potential risk factors (surgical indication, age, sex) with the incidence of postoperative hemorrhage was assessed.

## Materials and methods

**Fig. 1:**
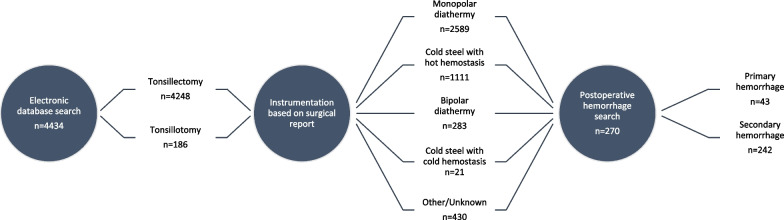
Data collection

The study was conducted in the Department of Otorhinolaryngology of Turku University Hospital, Turku, Finland. The study protocol was accepted by the institutional review board (permit number T06/040/21). In this retrospective study we analyzed the medical records of 4434 patients, who underwent tonsil surgery between 2012 and 2018 in the Hospital District of Southwest Finland, which is a non-insurance based public healthcare unit. Approximately 15% of all tonsil surgeries in Finland are performed in private healthcare units. Auria Clinical Informatics was used to collect data from medical records by searching for Nordic classification codes of surgical procedures: tonsillectomy (EMB10), tonsillotomy (EMB15), adenotonsillectomy (EMB20) and adenoidectomy (EMB30). The process of gathering data is presented in Fig. 1.

### General characteristics

The patients’ age, sex, indication for surgery, surgical method, surgical time, and surgical instrument were collected and divided into groups. The surgical method groups were TE and TT either with or without adenoidectomy. The indication groups were tonsillitis (recurrent or chronic), upper airway obstruction (tonsillar hypertrophy, obstructive sleep apnea, snoring, mouth breathing), peritonsillar abscess, tumor, or other (periodic fever, enlarged lymph nodes, neck mass, dysphagia, other chronic diseases of tonsils and adenoids, dentofacial anomaly). The age groups were ≤ 15 years and > 15 years.

### Surgical instruments

The instruments used for both dissection and hemostasis were retrieved from each patient’s surgical reports. The cold instruments were a knife, a tonsil elevator, scissors, and a microdebrider for dissection. For cold hemostasis, compression, packs, sutures, and epinephrine were used. The hot instruments were monopolar diathermy, bipolar diathermy (scissors or forceps), coblation, laser and radiofrequency. The surgical instruments were divided into groups according to the above: cold steel dissection with cold hemostasis, cold steel dissection with hot hemostasis, monopolar diathermy for both dissection and hemostasis, bipolar diathermy for both dissection and hemostasis, and unknown/other. Coblation (*n* = 4), laser (*n* = 1) and radiofrequency (*n* = 1) were classified as other due to their small number. The instrument was classified as unknown if it was not clear in the surgical report.

### Postoperative hemorrhage

Patients with a postoperative hemorrhage were sought using the ICD-10 diagnostic code T81.0 (Hemorrhage and hematoma complicating a procedure) and by an open word search, for example “bleed-”. Patients’ medical records were accessed for detailed evaluation. A postoperative hemorrhage was defined as any recorded bleeding episode requiring medical contact or intervention. Primary and secondary bleeding were defined as any bleeding that occurred within 24 h of surgery and between 1 and 28 days after surgery, respectively.

The bleeding complications were divided into groups by hemorrhage grades, which were modified from Windfuhr and Seehafer’s classification [15]: (1) Contact by phone, (2) Bleeding had stopped by itself before an examination, (3) Bleeding was treated at the clinic under local anesthesia, (4) Bleeding was treated in the operating room under general anesthesia. If a patient had more than one episode of bleeding, it was classified according to the bleeding requiring the most intervention.

### Statistics

Statistical analyses were performed using SPSS software (version 28.0, IBM Corp., Armonk, NY, USA). Binary logistic regression was used to analyze the risk factors of postoperative hemorrhage. Univariate analysis of variance (ANOVA) and post-hoc multiple comparisons with Bonferroni correction analysis were used to compare surgical time in different instrument groups. For decision tree analysis of postoperative hemorrhage risk factors, each reported putative risk factor was included in the independent variables and the CHAID method was used, setting the p-value criterion for splitting at 0.05. For analysis of instrumentation effects, an instrumentation variable was forced in the analysis. For all analyses, a p-value of < 0.05 was considered significant.

## Results

### Descriptive data

A total of 4434 patients underwent tonsil surgery between the years 2012–2018 in the Hospital District of Southwest Finland. Of these surgeries, 95.8% (n = 4248) were tonsillectomies and 4.2% (n = 186) tonsillotomies. Adenoidectomy was performed in 865 of these cases and 92.6% of adenoidectomy patients were aged ≤ 15 years.

**Fig. 2:**
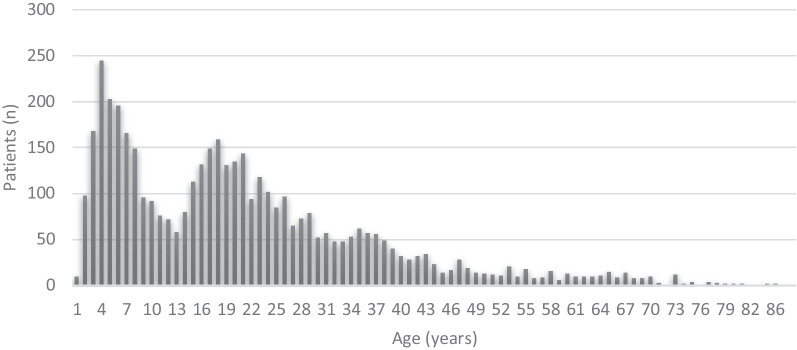
The distribution of age

Of all the patients, 57.6% were female and 58.9% were older than 15 years. The median age at the time of the operation was 18.0 years (range 1–91 years). The distribution of age is shown in Fig. 2. The main indications for tonsil surgery were tonsillitis in 49.7% and upper airway obstruction in 37.3%. The descriptive data are presented in Table 1.

**Table 1:** Patient and postoperative hemorrhage data

Variables	Patients	All postoperative hemorrhages	Primary hemorrhages	Secondary hemorrhages
Total number (*n* (%))	4434	270 (6.1)	43 (1.0)	242 (5.5)
Age				
Mean	21.0	24.1	22.9	24.6
≤ 15 years (*n* (%))	1822 (41.1)	60 (3.3)	11 (0.6)	50 (2.7)
> 15 years (*n* (%))	2612 (58.9)	210 (8.0)	32 (1.2)	192 (7.4)
Sex (*n* (%))				
Female	2553 (57.6)	141 (5.5)	25 (1.0)	125 (4.9)
Male	1881 (42.4)	129 (6.9)	18 (1.0)	117 (6.2)
Surgical method (*n* (%))				
TE ± A	4248 (95.8)	266 (6.3)	43 (1.0)	238 (5.6)
TT ± A	186 (4.2)	4 (2.2)	0 (0.0)	4 (2.2)
TE/TT + A	865 (19.5)	20 (2.3)	5 (0.6)	15 (1.7)
Indication (*n* (%))				
Tonsillitis	2202 (49.7)	177 (8.0)	26 (1.2)	163 (7.4)
Obstruction	1654 (37.3)	65 (3.9)	13 (0.8)	55 (3.3)
Abscess	428 (9.7)	22 (5.1)	2 (0.5)	20 (4.7)
Tumor	43 (1.0)	2 (4.7)	1 (2.3)	1 (2.3)
Other	107 (2.4)	4 (3.7)	1 (0.9)	3 (2.8)
Surgical instrument (*n* (%))				
Cold steel with cold hemostasis	21 (0.5)	2 (9.5)	0 (0.0)	2 (9.5)
Cold steel with hot hemostasis	1111 (25.1)	66 (5.9)	15 (1.4)	59 (5.3)
Monopolar diathermy	2589 (58.4)	159 (6.1)	20 (0.8)	145 (5.6)
Bipolar diathermy	283 (6.4)	23 (8.1)	3 (1.1)	21 (7.4)
Unknown/other	430 (9.7)	20 (4.7)	5 (1.2)	15 (3.5)
Surgical time (h:min)	0:28	0:26	0:28	0:26

*TE ± A*  tonsillectomy with or without adenoidectomy, *TT ± A* tonsillotomy with or without adenoidectomy, *TE/TT + A* tonsillectomy or tonsillotomy with adenoidectomy

The most frequently used surgical instrument was monopolar diathermy (58.4%). Cold steel dissection with hot hemostasis was used in 25.1% of the cases and bipolar diathermy in 6.4% (Table 1). Cold steel dissection with cold hemostasis was only used for 21 patients and was therefore excluded from further analysis. Cases with instruments defined as ‘unknown’ (n = 424) and ‘other’ (n = 6), were excluded from further analysis. In the cold steel dissection with hot hemostasis group, the hemostasis was mainly (96.7%) performed with monopolar diathermy.

### Postoperative hemorrhage

There was a total of 270 (6.1%) postoperative hemorrhages in the tonsil surgeries. The hemorrhage rate in the TE group was 6.3% (n = 266) and in the TT group 2.2% (n = 4). The hemorrhage rates for primary and secondary bleeding were 1.0% and 5.5%, respectively. In patients aged > 15 years, the bleeding rate was 8.0%, and in patients aged ≤ 15 years, 3.3% (Table 1).

Most commonly, the bleeding needed treatment at the clinic under local anesthesia (63.3%), usually by compression with epinephrine-soaked gauze or suction cautery. These patients were mainly older than 15 years (88.9%). Grade 4 bleedings covered 12.6% of all the bleeding patients, the overall hemorrhage rate being 0.8% in this group. The hemorrhage grades in both age groups are presented in Table 2.

**Table 2:** Hemorrhage grades in different age groups

Hemorrhage grade		Total (% of 4434 patients)	≤ 15 years old (% of 1822 patients)	> 15 years old (% of 2612 patients)
1	Contact by phone	8 (0.2)	3 (0.2)	5 (0.2)
2	Bleeding had stopped by itself before an examination	57 (1.3)	21 (1.2)	36 (1.4)
3	Bleeding treated at the clinic under local anesthesia	171 (3.9)	19 (1.0)	152 (5.8)
4	Bleeding treated in the operating room under general anesthesia	34 (0.8)	17 (0.9)	17 (0.7)
	In total	270 (6.1)	60 (3.3)	210 (8.0)

### Surgical time

The overall average surgical time was 28.7 min. In the cold steel with hot hemostasis group, it was 30.7 min, in the monopolar group 27.3 min, and in the bipolar group 29.9 min. ANOVA demonstrated a significant difference between groups. Post-hoc multiple comparisons with Bonferroni correction analysis showed monopolar being a faster technique than cold steel with hot hemostasis (p < 0.001) and bipolar diathermy (p = 0.047).

### Postoperative hemorrhage analyses

The logistic regression analyses of postoperative hemorrhage in different variable groups are shown in Table 3.

**Table 3:** Postoperative hemorrhage analyses with logistic regression

	All postoperative hemorrhages		Primary hemorrhages		Secondary hemorrhages	
	*OR* (95% CI)	*p-*value	*OR* (95% CI)	*p*-value	*OR* (95% *CI*)	*p*-value
Age						
≤ 15 years	1		1		1	
> 15 years	2.57 (1.92; 3.44)	< 0.001*	2.04 (1.03; 4.06)	0.042*	2.81(2.05; 3.86)	< 0.001*
Sex						
Female	1		1		1	
Male	1.26 (0.98; 1.61)	0.067	0.98 (0.53; 1.80)	0.940	1.29 (0.99; 1.67)	0.056
≤ 15 y sex						
≤ 15 years old female	1		1		1	
≤ 15 years old male	1.89 (1.12; 3.18)	0.017*	1.10 (0.34; 3.62)	0.874	2.20 (1.24; 3.93)	0.007*
Indication						
Tonsillitis	1		1		1	
Obstruction	0.47 (0.35; 0.63)	< 0.001*	0.66 (0.34; 1.29)	0.228	0.43 (0.32; 0.59)	< 0.001*
Abscess	0.62 (0.39; 0.98)	0.040*	0.39 (0.09; 1.66)	0.204	0.61 (0.38; 0.99)	0.044*
Tumor	0.56 (0.13; 2.33)	0.423	1.99 (0.26; 15.03)	0.504	0.30 (0.04; 2.18)	0.233
Other	0.44 (0.16; 1.22)	0.116	0.79 (0.11; 5.87)	0.817	0.36 (0.11; 1.15)	0.085
Indication ≤ 15 y TE						
Tonsillitis	1		1		1	
Obstruction	0.56 (0.32; 0.98)	0.043*	1.38 (0.29; 6.54)	0.683	0.44 (0.24; 0.81)	0.008*
Abscess	0.53 (0.12; 2.33)	0.402	0	1	0.56 (0.13; 2.47)	0.444
Tumor	0	1	0	1	0	1
Other	0.31 (0.04; 3.26)	0.259	3.26 (0.29; 36.55)	0.337	0	1
Surgical method						
TE ± A	1		1		1	
TT ± A	0.33 (0.12; 0.89)	0.029*	0	0.996	0.37 (0.14; 1.01)	0.051
TE/TT ± A						
TE/TT – A	1		1		1	
TE/TT + A	0.31 (0.20; 0.50)	< 0.001*	0.54 (0.21; 1.38)	0.197	0.26 (0.15; 0.44)	< 0.001*
≤ 15 y TE ± A						
≤ 15 years old TE – A	1		1		1	
≤ 15 years old TE + A	0.44 (0.24; 0.81)	0.008*	1.03 (0.31; 3.40)	0.958	0.34 (0.17; 0.68)	0.002*
Surgical instrument						
CS with hot hemostasis	1		1		1	
Monopolar diathermy	1.04 (0.77; 1.39)	0.815	0.57 (0.29; 1.12)	0.100	1.06 (0.78; 1.44)	0.723
Bipolar diathermy	1.40 (0.86; 2.30)	0.181	0.78 (0.23; 2.72)	0.700	1.43 (0.85; 2.40)	0.175
Surgical instrument for TE						
Bipolar diathermy	1		1		1	
CS with hot hemostasis	0.57 (0.35; 0.94)	0.027*	1.04 (0.30; 3.62)	0.950	0.56 (0.33; 0.94)	0.029*
Monopolar	0.60 (0.38; 0.96)	0.031*	0.61 (0.18; 2.08)	0.434	0.60 (0.37; 0.98)	0.039*
Surgical instrument for TE						
CS with hot hemostasis	1		1		1	
Monopolar diathermy	1.06 (0.79; 1.42)	0.719	0.59 (0.30; 1.16)	0.125	1.08 (0.79; 1.47)	0.646
Bipolar diathermy	1.75 (1.07; 2.88)	0.027*	0.96 (0.28; 3.35)	0.950	1.78 (1.06; 3.00)	0.029*

*OR* odds ratio, *CI* confidence interval, *TE* tonsillectomy, *TT* tonsillotomy, *A* adenoidectomy, *CS* cold steel; ±  with or without

*Statistically significant

### Postoperative hemorrhage analyses by patient characteristics

Compared to patients aged > 15 years, patients aged ≤ 15 years had a significantly lower risk both for a primary and for a secondary hemorrhage. In patients aged ≤ 15 years, the risk for secondary hemorrhage increased with age (OR = 1.12; 95% CI 1.05–1.20; p < 0.001), but in patients aged > 15 years, the risk for secondary hemorrhage decreased with age (OR = 0.99; 95% CI 0.98–1.00; p = 0.039).

Male sex was a statistically significant risk factor of postoperative hemorrhage in patients aged ≤ 15 years but not in patients aged > 15 years.

Analyzing surgical indications, the tonsillitis group had a significantly higher total and secondary hemorrhage rates than the obstruction group and the abscess group. Regarding primary hemorrhages, there were no statistically significant differences.

Tonsillotomy was performed almost exclusively for obstructive indications for patients aged ≤ 15 years, and thus, the analysis was repeated with the inclusion of only TE patients. The higher risk of total and secondary hemorrhage in tonsillitis patients compared to obstruction and abscess patients remained significant in TE patients. Further, the analysis was repeated separately for the age groups in TE patients. In TE patients aged ≤ 15 years, there was a higher risk for total and secondary hemorrhage in the tonsillitis group than in the obstruction group. In patients older than 15 years, there were no significant differences between the indications.

### Postoperative hemorrhage analyses by surgical methods

The postoperative hemorrhage rate in the TE group was 6.3% and in the TT group 2.2%. The secondary hemorrhage rate for TE was higher (5.6%) compared to the risk in the TT group (2.2%), but the difference was not statistically significant.

If adenoidectomy was performed with TE or TT, there was a lower risk for postoperative bleeding. The reduction in risk remained significant when only the TE patients aged ≤ 15 years were analyzed.

### Postoperative hemorrhage analyses by surgical instruments

Comparing different instruments including both TE and TT patients, there were no statistically significant differences in the primary, secondary or total hemorrhage rates.

The difference between instruments was analyzed separately for the TT and TE groups. In the TE group, the risk for secondary hemorrhage was higher with bipolar diathermy compared to cold steel with hot hemostasis and compared to monopolar. Between the monopolar and the cold steel with hot hemostasis groups, the differences were statistically non-significant regarding the bleeding rates in TE patients (Table 3). In the TT group, there were no significant differences between instruments.

### Primary and secondary bleeding

Primary bleeding was associated with a higher risk of secondary bleeding (OR = 9.83; 95% CI 5.18–18.66; p < 0.001).

### Surgical time

Surgical time was not significantly associated with postoperative bleeding.

### Decision tree analysis

**Fig. 3:**
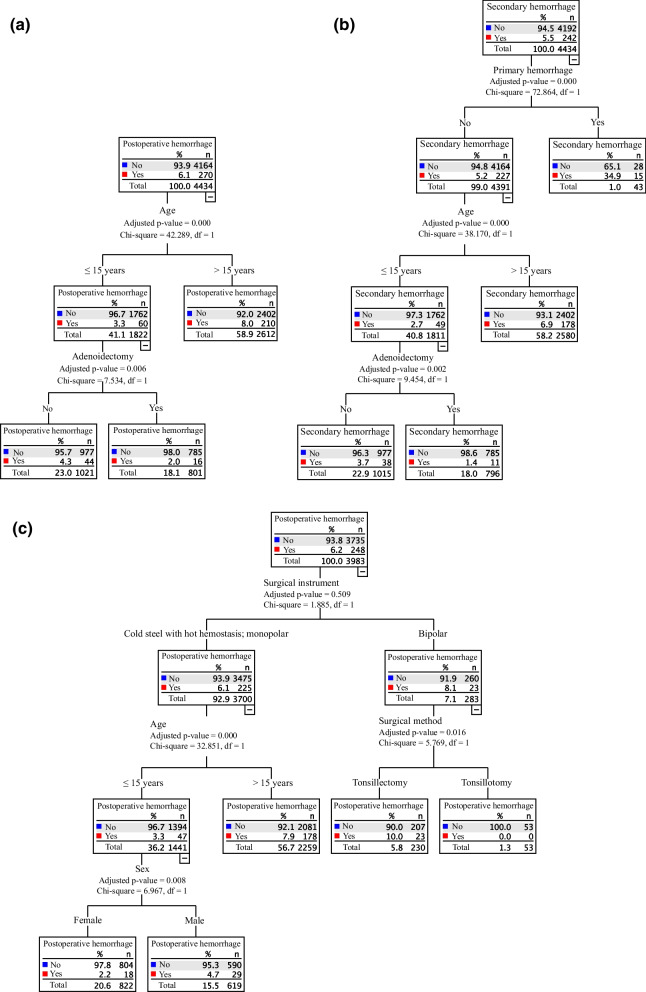
**a** The risk factors for postoperative hemorrhage were analyzed using a decision tree analysis. **b** The risk factors for secondary hemorrhage were analyzed using a decision tree analysis. **c** The effect of surgical instrumentation was analyzed using a decision tree analysis

The decision tree confirming the significance of patient age and adenoidectomy in predicting postoperative hemorrhage is shown in Fig. 3a. In the analysis of secondary bleeding risk factors, the significance of primary bleeding as a risk factor for secondary bleeding was confirmed (Fig. 3b).

To analyze the effect of instrumentation on the decision tree, the instrumentation variable was forced in the analysis, demonstrating a similar decision tree between monopolar diathermy and cold steel with hot hemostasis, whereas the risk factors lost significance in the decision tree of bipolar diathermy (Fig. 3c).

## Discussion

In the TE group, the risk for a secondary hemorrhage was higher with bipolar diathermy compared to cold steel with hot hemostasis and compared to monopolar. The monopolar technique was faster than the cold steel with hot hemostasis technique and the bipolar technique. Patients aged over 15 years had a greater risk for primary and secondary hemorrhage. The total incidence of postoperative hemorrhage was higher in TE patients than in TT patients. Tonsillitis as an indication, primary hemorrhage, TE/TT without adenoidectomy, and male sex in patients aged ≤ 15 years increased the risk for a secondary hemorrhage.

Age has been a notable risk factor for postoperative hemorrhage in many studies [9, 10, 16–18]. Our findings support this, as patients older than 15 years had an increased risk for both primary and secondary bleeding. However, according to our study, increasing age does not directly translate to an increased bleeding risk, since in patients over 15 years, there was a significant trend for decreasing risk of postoperative hemorrhage.

There was a higher risk for postoperative hemorrhage in the TE group compared to the TT group, as has been well-documented in several earlier studies [19–22]. The overall bleeding rates for TE/TT were 6.3/2.2%. The highest rates were in the bipolar group (8.1%), the > 15 years group (8.0%) and the tonsillitis group (8.0%), these being still moderate compared to some studies [5, 7, 11, 17]. Furthermore, the rate for grade 4 bleedings was relatively low (0.8%) [11]. The variability of the patients and definitions of postoperative bleeding might explain these differences.

When adenoidectomy was performed with TE or TT, there was a decreased risk for postoperative hemorrhage than in the group of TE/TT without adenoidectomy. Similar results have been reported previously [22]. However, there are also opposite results [18]. In the TE/TT with adenoidectomy group, the mean surgical time was 31.8 min and without adenoidectomy it was 28.0 min. It is possible that the hemostasis is checked more carefully when adenoidectomy is performed in conjunction with tonsil surgery and the surgical time is longer. Primary bleeding was significantly associated with higher risk for secondary bleeding, which is in line with other studies [7, 11, 23].

Tonsillitis patients are more likely to suffer from postoperative hemorrhage than obstruction patients [9, 24–26]. Our findings support these results, especially in patients aged ≤ 15 years. In addition, patients with an abscess had a lower risk for postoperative bleeding compared to tonsillitis patients as reported before [11].

Several studies have shown male patients as being at a greater risk of postoperative bleeding [3, 10, 11, 17], although other studies have not found a significant difference between male and female risk [27, 28]. In our study, males aged ≤ 15 years had a higher risk for secondary hemorrhage than females in the same age group. However, in patients older than 15 years, there seemed to be no difference between sex.

In our study, in TE patients, bipolar diathermy increased the risk for secondary hemorrhage compared to monopolar and cold steel with hot hemostasis. In a large register study of Söderman et al., all hot techniques including cold steel with hot hemostasis resulted in a higher risk for late post-tonsillectomy hemorrhage compared to cold steel with cold hemostasis [7]. The hot techniques included bipolar scissors, coblation and ultrascision. They also found that bipolar and ultrascision techniques increased the risk for late post-tonsillectomy hemorrhage compared to cold steel with hot hemostasis, which is in line with our findings. In a randomized controlled trial of 245 patients, the overall hemorrhage rate was higher with bipolar diathermy than with cold dissection (12.1% versus 7.7%), but the difference did not reach statistical significance [29]. In their study, the hemostasis in the cold dissection group was achieved with ties in the lower poles and with sparing use of bipolar diathermy. In a prospective audit study by Lowe et al., the hemorrhage rates for hot techniques and cold steel with hot hemostasis were significantly higher than in the cold steel with cold hemostasis group [9]. Monopolar diathermy was included in their study alongside bipolar diathermy and coblation as hot techniques. Apart from this study, the risk of postoperative hemorrhage associated with monopolar diathermy and comparing it with other common techniques, has not been as extensively studied even though it is a commonly used instrument. Our study’s detailed information on the monopolar, bipolar, and cold steel with hot hemostasis techniques is not present in the study by Sarny et al. [11]. Not only does our study complement their study, it also provides additional unique information to the literature. Our results support previous findings that bipolar diathermy increases the risk for post-tonsillectomy hemorrhage. However, there seems to be no statistical difference in the bleeding rates when comparing monopolar diathermy and the cold steel with hot hemostasis technique.

In the Hospital District of Southwest Finland, monopolar diathermy was the most used instrument between the years 2012–2018, although most surgeons in training are first taught to operate with the cold steel technique, before being allowed to use hot techniques. Monopolar diathermy is considered to be easy to use, cost-effective, fast, and a relatively safe technique [19, 25, 26]. In our study, the surgical time was slightly shorter with the monopolar technique compared to the cold steel with hot hemostasis and the bipolar techniques. Considering our findings on monopolar diathermy, it seems safe to continue using this instrumentation for TE.

### Limitations

The limitations of this study are related to its retrospective and single-institution study design. In Finland, tonsil surgeries are mostly performed as day-surgeries. We advise patients that minor bleedings are common and can be observed at home for a short time. Thus, patients may not have contacted the hospital for all minor bleedings. It is also possible that some of the phone contacts were not recorded. We advise patients to contact our clinic directly in case of postoperative bleeding. Because we are the only hospital with ear, nose and throat specialists on-call in our region, it is likely that the patients with postoperative bleeding were treated in our hospital. However, we cannot rule out that some patients may have attended in another hospital for treatment. In addition, there might have been patients with postoperative bleeding that the search for the diagnostic code T81.0 or the open words could not find.

As stated above, earlier studies have shown cold steel with cold hemostasis lowers the risk for secondary hemorrhage compared to hot techniques [7, 9, 12], but we could not reliably analyze this due to the small number of cold steel with cold hemostasis techniques in our data. Due to the retrospective study design, we cannot rule out that any hot techniques were not used for dissection in the cold steel with hot hemostasis group. However, the surgical reports were thoroughly reviewed, and they include at our institution the surgical technique and the applied instrumentation almost unequivocally. In addition, there were relatively few tonsillotomies in this data, but we chose to include the TT patients due to their increasing number in recent years. In the literature, evidence of greater pain has been presented after the use of diathermy versus cold dissection in tonsillectomy [14]. Unfortunately, we could not analyze the level of postoperative pain from our retrospective data. Pain is a significant factor in tonsil surgeries, and it is important to treat it well [30]. In this study, we did not analyze whether the experience of the surgeons was associated with postoperative hemorrhage. Recent studies have shown, that more experienced surgeons perform tonsillectomy more quickly, however, no differences have been detected in the postoperative bleeding rates between residents and senior surgeons [31, 32].

## Conclusions

In this study, bipolar diathermy was associated with an increased risk for secondary bleedings compared to both monopolar diathermy and the cold steel with hot hemostasis technique in tonsillectomy patients. Monopolar diathermy did not differ from the cold steel with hot hemostasis technique regarding the bleeding rates.

In tonsil surgeries, age is a risk factor for primary and secondary hemorrhage. Recurrent or chronic tonsillitis as an indication and primary hemorrhage increase the risk for a secondary hemorrhage. If adenoidectomy is performed with tonsil surgery, it might decrease the risk for postoperative hemorrhage. In tonsillotomy the incidence of postoperative bleeding is lower than in tonsillectomy.

## Data Availability

Data of the patients are presented in Table 1, Figs. 1, and 2. Statistical analyses were performed using SPSS software (version 28.0, IBM Corp., Armonk, NY, USA). Statistical analyses are presented in Table 3 and Fig. 3.

## References

[1] Cullen KA, Hall MJ, Golosinskiy A (2009) Ambulatory surgery in the United States, 2006. Natl Health Stat Report, pp 1–25 19294964

[2] Windfuhr JP, Werner JA (2013). Tonsillotomy: it’s time to clarify the facts. Eur Arch Otorhinolaryngol.

[3] Windfuhr JP, Chen YS, Remmert S (2005). Hemorrhage following tonsillectomy and adenoidectomy in 15,218 patients. Otolaryngol-Head Neck Surg.

[4] Windfuhr JP (2013). Serious complications following tonsillectomy: how frequent are they really?. ORL J Otorhinolaryngol Relat Spec.

[5] Tolska HK, Takala A, Pitkäniemi J, Jero J (2013). Post-tonsillectomy haemorrhage more common than previously described—an institutional chart review. Acta Otolaryngol.

[6] Windfuhr JP, Savva K, Dahm JD, Werner JA (2015). Tonsillotomy: facts and fiction. Eur Arch Otorhinolaryngol.

[7] Söderman A-CH, Odhagen E, Ericsson E, Hemlin C, Hultcrantz E, Sunnergren O (2015). Post-tonsillectomy haemorrhage rates are related to technique for dissection and for haemostasis. An analysis of 15734 patients in the National Tonsil Surgery Register in Sweden. Clin Otolaryngol.

[8] Lundström F, Stalfors J, Østvoll E, Sunnergren O (2020). Practice, complications and outcome in Swedish tonsil surgery 2009–2018. An observational longitudinal national cohort study. Acta Otolaryngol.

[9] David Lowe F, Jan van der Meulen P, David Cromwell P, James Lewsey P, Lynn Copley MS, John Browne P (2007). Key messages from the national prospective tonsillectomy audit 2007. Laryngoscope.

[10] Tomkinson A, Harrison W, Owens D, Harris S, McClure V, Temple M (2011). Risk factors for postoperative hemorrhage following tonsillectomy. Laryngoscope.

[11] Sarny S, Ossimitz G, Habermann W, Stammberger H (2011). Hemorrhage following tonsil surgery: a multicenter prospective study. Laryngoscope.

[12] Morris S, Martin T, Lewis S (2018). Cold/cold vs. bipolar dissection tonsillectomy: a surgeon-controlled study of 400 cases. Clin Otolaryngol.

[13] Setabutr D, Adil EA, Adil TK, Carr MM (2011). Emerging trends in tonsillectomy. Otolaryngol-Head Neck Surg.

[14] Pinder DK, Wilson H, Hilton MP (2011). Dissection versus diathermy for tonsillectomy. Cochrane Database Syst Rev.

[15] Windfuhr S (2001). Classification of haemorrhage following tonsillectomy. J Laryngol Otol.

[16] Harju T, Numminen J (2017). Risk factors for secondary post-tonsillectomy haemorrhage following tonsillectomy with bipolar scissors: four-year retrospective cohort study. J Laryngol Otol.

[17] Mueller J, Boeger D, Buentzel J, Esser D, Hoffmann K, Jecker P (2015). Population-based analysis of tonsil surgery and postoperative hemorrhage. Eur Arch Otorhinolaryngol.

[18] Gonçalves AI, Rato C, de Vilhena D, Duarte D, Lopes G, Trigueiros N (2020). Evaluation of post-tonsillectomy hemorrhage and assessment of risk factors. Eur Arch Otorhinolaryngol.

[19] Sakki AJ, Mäkinen LK, Kanerva M, Nokso-Koivisto J (2021). Monopolar tonsillotomy versus cold dissection tonsillectomy in children: prospective study on postoperative recovery. Int J Pediatr Otorhinolaryngol.

[20] Wong Chung JERE, van Benthem PPG, Blom HM (2018). Tonsillotomy versus tonsillectomy in adults suffering from tonsil-related afflictions: a systematic review. Acta Otolaryngol.

[21] Sarny S, Habermann W, Ossimitz G, Stammberger H (2013). What lessons can be learned from the Austrian events?. ORL.

[22] Odhagen E, Stalfors J, Sunnergren O (2019). Morbidity after pediatric tonsillotomy versus tonsillectomy: a population-based cohort study. Laryngoscope.

[23] Attner P, Haraldsson P-O, Hemlin C, Hessén Söderman A-C (2009). A 4-year consecutive study of post-tonsillectomy haemorrhage. ORL.

[24] Lane JC, Dworkin-Valenti J, Chiodo L, Haupert M (2016). Postoperative tonsillectomy bleeding complications in children: a comparison of three surgical techniques. Int J Pediatr Otorhinolaryngol.

[25] Thottam PJ, Christenson JR, Cohen DS, Metz CM, Saraiya SS, Haupert MS (2015). The utility of common surgical instruments for pediatric adenotonsillectomy. Laryngoscope.

[26] Lou Z, Lou Z, Lv T, Chen Z (2022). A prospective, randomized, single-blind study comparing coblation and monopolar extracapsular tonsillectomy. Laryngoscope Investig Otolaryngol.

[27] Schrock A, Send T, Heukamp L, Gerstner AO, Bootz F, Jakob M (2009). The role of histology and other risk factors for post-tonsillectomy haemorrhage. Eur Arch Otorhinolaryngol.

[28] Wei JL, Beatty CW, Gustafson RO (2000). Evaluation of Posttonsillectomy Hemorrhage and Risk Factors. Otolaryngol-Head Neck Surg.

[29] Haddow K, Montague M-L, Hussain SSM (2006). Post-tonsillectomy haemorrhage: a prospective, randomized, controlled clinical trial of cold dissection versus bipolar diathermy dissection. J Laryngol Otol.

[30] Aldamluji N, Burgess A, Pogatzki-Zahn E, Raeder J, Beloeil H, Albrecht E (2021). PROSPECT guideline for tonsillectomy: systematic review and procedure-specific postoperative pain management recommendations. Anaesthesia.

[31] Naara S, Aronov M, Gil Z, Gordin A (2021). Can tonsillectomy be safely performed by residents? A comparative retrospective study. Ann Otol Rhinol Laryngol.

[32] Leader BA, Wiebracht ND, Meinzen-Derr J, Ishman SL (2020). The impact of resident involvement on tonsillectomy outcomes and surgical time. Laryngoscope.

